# Therapeutic Potential of Enoxaparin in Lichen Planus: Exploring Reasons for Inconsistent Reports

**DOI:** 10.3389/fphar.2018.00586

**Published:** 2018-06-05

**Authors:** Rahul P. Patel, Madhur D. Shastri, Long Chiau Ming, Syed Tabish R. Zaidi, Gregory M. Peterson

**Affiliations:** ^1^Pharmacy, School of Medicine, University of Tasmania, Hobart, TAS, Australia; ^2^School of Health Sciences, University of Tasmania, Launceston, TAS, Australia; ^3^School of Pharmacy, KPJ Healthcare University College, Nilai, Malaysia; ^4^Vector-borne Diseases Research Group (VERDI), Pharmaceutical and Life Sciences CoRe, Universiti Teknologi MARA, Shah Alam, Malaysia

**Keywords:** anti-inflammatory, batch-to-batch variation, chromatography, enoxaparin, heparin, lichen planus, non-anticoagulant, response

## Abstract

Lichen planus (LP) is an uncommon mucocutaneous inflammatory condition, that is immunologically mediated, typically pruritic and often recurs. The currently advocated therapies are either not highly effective or associated with severe side effects. Enoxaparin, a widely used anticoagulant, is composed of both anticoagulant and non-anticoagulant fragments. Enoxaparin is reported to have anti-inflammatory properties and it was found to be effective in LP. However, the results from clinical studies have varied substantially and, therefore, the clinical role of enoxaparin in LP remains uncertain. This review focuses on potential reasons for the reported inconsistent outcomes, as well as proposing solutions; these include identifying batch-to-batch inconsistency in the composition of enoxaparin. The potential therapeutic value of enoxaparin in LP must be explored using well-designed clinical trials, combined with experimental studies that focus on identifying the anti-inflammatory fragments of enoxaparin and elucidating the mechanism of action of these non-anticoagulant fragments.

## Introduction

Lichen planus (LP) is a mucocutaneous inflammatory condition that can present with a variety of clinical manifestations. It mainly affects the skin, nails, scalp, and oral and genital mucous membranes. It is characterized by itchy, purplish, polygonal, flat-topped papules with lacy white lines (Wickham’s striae) (Gorouhi et al., 2014; Weston and Payette, 2015). The precise prevalence of LP is unknown but is estimated to be between 0.22 and 5% worldwide (Gorouhi et al., 2014). It can be associated with hepatitis C viral infection (Shengyuan et al., 2009). Without therapy, the skin lesions usually resolve over 6–18 months, although in up to 20% of patients relapse occurs in the same area as the initial episode (Gorouhi et al., 2014). Chronic disease is more likely with oral LP.

The etiology of LP is yet to be fully understood, but an immunological abnormality is believed to play an important role, notably involving antigen-presenting cells. Evidence so far suggests that LP is associated with T-cell mediated inflammation, in particular increased expression of heparanase and activation of keratinocytes (Daoud and Pittelkow, 2012). Patients with LP are reported to have elevated levels of several inflammatory mediators (tumor necrosis factor alpha (TNF-α), interleukin (IL)-2, IL-4, IL-6, IL-10 and basic fibroblast growth factor), correlating with the severity of the condition (Simark-Mattsson et al., 1999; Pezelj-Ribaric et al., 2004; Gorugantula et al., 2012; Kaur and Jacobs, 2015). These inflammatory mediators stimulate the accumulation of T cells in the epidermis, ultimately resulting in destruction of the epidermis, referred to as a lichenoid tissue reaction.

A wide range of therapeutic approaches, including topical, systemic and intralesional corticosteroids, antihistamines, calcineurin inhibitors (e.g., cyclosporine, pimecrolimus, sirolimus, and tacrolimus), sulfasalazine, systemic and topical retinoids, aloe vera, and extracorporeal photo-chemotherapy, has been used for the treatment of the various forms of LP (Farhi and Dupin, 2010). However, the lack of evidence for efficacy of the currently advocated pharmacological agents, as well as their potential for significant side effects, can make the treatment frustrating for both clinicians and patients (Farhi and Dupin, 2010). Therefore, the search for safer and more effective modalities for the management of LP continues. Enoxaparin, a type of glycosaminoglycan, has attracted much interest among researchers due to its reported anti-inflammatory properties.

## Enoxaparin

**FIGURE 1:**
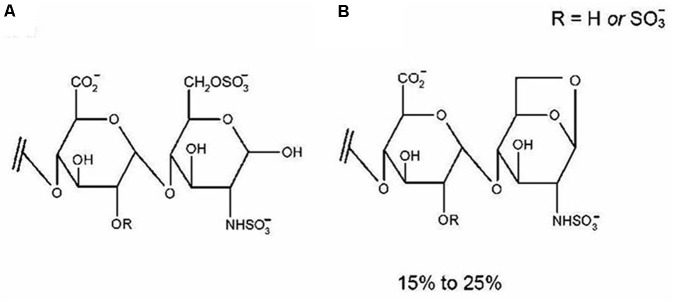
The disaccharide contains two residues of uronic acid and d-glucosamine linked by a 1→4 glycosidic linkage **(A)**. Approximately 15–25% of enoxaparin oligosaccharides contain 1,6-anhydro groups at their reducing end **(B)** as a result of chemical b-eliminative cleavage of unfractionated heparin. Reproduced with permission (Patel et al., 2009c).

Enoxaparin, the first low-molecular-weight heparin (LMWH) approved by the Food and Drug Administration, has an average molecular weight of 4500 Da. Enoxaparin has largely replaced unfractionated heparin in clinical practice due to its fewer side effects, higher anti-factor (AFXa) activity and more predictable dose-response relationship (Dekker et al., 2016). As shown in **Figure 1A**, it is a highly negatively charged linear polysaccharide composed of repeating disaccharide units of D-glucosamine and uronic acid linked by 1→4 glycosidic bonds (Perkins et al., 2014). Enoxaparin is obtained by controlled chemical eliminative cleavage of the benzyl ester of heparin with alkaline treatment. This process results in the formation of LMW chains, of which typically 15–25% contain a 1,6-anhydroglucosamine at the reducing end (**Figure 1B**) (Perkins et al., 2014).

### Non-anticoagulant Fragments of Enoxaparin and Inflammation

Enoxaparin is known to have a wide range of biological activities. It is a mixture of highly negatively charged, structurally complex fragments (dp2 – two saccharides to approximately dp22 – twenty two saccharides) with undefined structure and inherent variability. Enoxaparin possesses both anticoagulant and non-anticoagulant fragments, with substantial evidence that the latter fragments are accountable for the reported anti-inflammatory effects of enoxaparin. For example, in an *in vitro* study, lung epithelial cells were stimulated to release two inflammatory cytokines, IL-6 and IL-8. Stimulated cells were treated with either enoxaparin or its various fragments. The disaccharide fragments (dp2) without anticoagulant activity were found to be responsible for the anti-inflammatory effect of enoxaparin (Shastri et al., 2015b). In another study, peripheral blood mononuclear cells from patients with allergic inflammation were activated in the presence or absence of enoxaparin fragments before measuring the levels of inflammatory cytokines (Shastri et al., 2015c). Two fragments of enoxaparin without anticoagulant activity were found to be responsible for the inhibition of cytokine secretion. A disaccharide fragment (dp2) inhibited the release of IL-4, IL-5, IL-12, and TNF-α by more than 57%, while a tetrasaccharide fragment (dp4) inhibited the release of these cytokines by 68%.

Lean et al. (2014) identified various fragments of enoxaparin with pro- and anti-proliferative effects. The authors treated the human colonic epithelial cancer cells in the presence of enoxaparin or its various fragments. Interestingly, the smallest fragment of enoxaparin (dp2), devoid of any anticoagulant activity, showed the strongest anti-proliferative effect (Lean et al., 2014). Shastri et al. (2013) investigated the ability of enoxaparin-derived fragments to inhibit nitric oxide production by lipopolysaccharide-activated macrophages. The disaccharide fragment of enoxaparin did not exhibit any anticoagulant activity but it reduced the production of nitric oxide by 50%. In a preclinical study, male C57BL/6 mice with chemically induced intestinal inflammation were treated with various fragments of enoxaparin. The tetrasaccharide fragments (dp4) of enoxaparin prevented the increase of relative colon weight, and the hexasaccharides (dp6) selectively reduced shortening of the colon (Lean et al., 2016). The authors reported that the identified active tetrasaccharides did not exert anticoagulant activity and the hexasaccharides had a low risk of bleeding, as the measured anticoagulant activity was reduced 10-fold compared to parent enoxaparin.

### Clinical Use of Enoxaparin in LP

Topical and intralesional corticosteroids are the current first-line therapy for LP (Lebwohl et al., 2017). However, large scale randomized clinical trials are still warranted to establish the efficacy of corticosteroids, as well as other available modalities for different variants of LP (Atzmony et al., 2016). At present, drug treatments for LP are either not completely effective and/or associated with severe side effects (Atzmony et al., 2016). LP can be chronic and relapsing in nature, and is often resistant to the currently available pharmacological agents. Therefore, the search for more effective and safer therapeutic agents for the treatment of LP continues.

Enoxaparin is the only derivative of heparin that has been so far evaluated for its clinical efficacy in LP. The studies have reported mixed but encouraging clinical outcomes. Ingber et al. (1994) demonstrated that low subcutaneous doses of enoxaparin inhibited the elicitation of allergic contact dermatitis. This preliminary finding led to the investigation of enoxaparin in 11 patients with histopathologically proven LP (Hodak et al., 1998). Subcutaneous injection of 3 mg of enoxaparin once-weekly for either 4 or 6 weeks resulted in complete regression of the eruption, with residual post-inflammatory hyperpigmentation in more than 70% of patients (8 out of 11). On the basis of these encouraging early findings, the potential role of enoxaparin in LP was further explored by researchers internationally.

To date, one randomized clinical trial, and twelve small, open-labeled and non-randomized clinical studies have investigated the therapeutic efficacy of subcutaneously administered enoxaparin in LP (**Table 1**). Stefanidou et al. (1999) evaluated the efficacy of enoxaparin in 18 patients with various types of LP and reported complete remission in 61% of patients and marked improvement in a further 11%. Similarly, Pacheco and Kerdel (2001) reported marked improvement in 5 out of 7 patients with LP when treated with enoxaparin. In another study, patients with LP were treated with enoxaparin for 4–14 weeks and 21 of 24 patients achieved complete remission (Akdeniz et al., 2005). Similarly, once-weekly use of enoxaparin over a period of 20 weeks resulted in a dramatic improvement in visual analog scale assessments of pain and itch in 13 out of 15 patients unresponsive to topical or oral corticosteroid therapy (Ameen and Alfadhily, 2011). Yasar et al. (2011) reported complete remission of palmoplantar hyperkeratotic LP (HPLP), a relatively uncommon form of LP that is mostly resistant to conventional treatments, in 2 patients treated with enoxaparin for 3 months (Yasar et al., 2011). No recurrence of PHLP was noted in follow-ups performed over a period of 1 year. Khan et al. (2014) treated cutaneous LP with enoxaparin for 6 weeks. They reported disease improvement in 26 out of 31 patients. Ucmak and co-workers reported distinctive recovery of the disease in 71% of patients (15 out of 21) treated with enoxaparin for 12 weeks (Uçmak et al., 2012). Neville et al. (2007) demonstrated a sustained clinical response to enoxaparin in a patient with recalcitrant ulcerative LP resistant to oral corticosteroids. All of the reports above used 3 mg of subcutaneous enoxaparin once-weekly and reported that the therapy was well tolerated and was not associated with any major side effects.

**Table 1:** Studies investigating the clinical efficacy of subcutaneous enoxaparin in patients with lichen planus (LP).

Author	Year	Study design	Patients	No. of patients	Enoxaparin dose/duration	Previous treatment	Clinical effect	Side effects
Hodak et al.	1998	Pilot	Histopathologically proven LP with intense pruritus for 2–36 months. One patient had palmoplantar involvement and 4 had oral lesions	10	3 mg once weekly for 4 or 6 weeks	Topical corticosteroids and oral H1 blockers	Eight patients achieved a complete remission; one patient had marked clinical improvement; no clinical effect was observed in one patient	No side effects were observed
Stefanidou et al.	1999	Open-label	Hypertrophic LP, disseminated LP, disseminated and reticular oral LP, localized LP, localized and reticular oral LP, erosive oral LP or disseminated and erosive oral LP	18	3 mg once weekly for 6–13 weeks	Topical corticosteroids and/or systemic corticosteroids and/or cyclosporine and/or retinoids	11 of 18 patients achieved a complete remission; two achieved marked improvement; 5 patients showed no change	No side effects were observed
Pacheco and Kerdel	2001	Case series	Oral LP, lichen planopilaris, erosive genital LP, generalized eruptive LP	7	30 mg once a day for 6 months or once a week for 4, 6, or 12 weeks or once every other day for 1 or 4 months or once a week for 10 weeks	Topical and or systemic corticosteroids	5 of 7 patients experienced a marked improvement and 2 patients had no clinical improvement	No side effects were observed
Rai et al.	2002	Open-label	Histopathologically proven LP with or without associated oral lesions	10	3 mg once weekly for 6 weeks	Previously untreated patients	Complete remission was not seen in any patients. Clinical response was seen in only 2 of 10 patients	1 patient developed skin necrosis after the 4th injection and one patient developed multiple lesions over both lower and upper extremities with increased itching after the fourth injection
Ferahbas et al.	2003	Open-label	Histopathologically proven disseminated LP, disseminated with papular LP, localized LP, localized with papular LP, hypertrophic LP or localized with hypertrophic LP	7	5 mg once a week for 6 weeks	Topical and/or systemic corticosteroids, PUVA, sedatives and systemic anti-histamines	6 of 7 patients had no histological changes. 5 of 7 patients had no clinical improvement and itching decreased in 2 patients	No side effects were observed
Akdeniz et al.	2005	Open-label	Histopathologically proven LP with intense pruritus. Patients had either disseminated, disseminated with reticulated oral LP or hypertrophic LP	24	3 mg once a week for maximum 14 weeks	Topical and/or systemic corticosteroids	20 of 24 patients achieved a complete remission and 4 patients had no or minimal clinical effect	No side effects were observed
Neville et al.	2007	Case study	Patient with ulcerative LP and hepatitis C	1	30 mg biweekly for the first 2 weeks and then 3 mg biweekly for the next 4 weeks	Topical corticosteroid and topical immunosuppressant	Complete remission of the lesions and the patient was in remission for 18 months. A slight flare of LP on the palm was developed later on which was controlled with topical treatment of corticosteroid and immunosuppressant	No side effects were observed
Ameen and Alfadhily	2011	Open-label	Patients with recalcitrant LP	15	3 mg once a week for a period of 20 weeks	Topical and systemic corticosteroids	2 of 15 patients with mild form of LP had complete remission of the lesions. 13 of 15 patients with moderate or severe LP did not achieve complete remission. 13 of 15 patients reported significant reduction in itching	No side effects were observed
Yasar et al.	2011	Case series	Palmoplantar hyperkeratotic variant of LP	2	3 mg once a week for 12 weeks	Not specified	Skin lesions were healed in both the patients but oral mucosal lesions remained stable	No side effects were observed
Ucmak et al.	2012	Open-label	LP - Type of LP not specified	21	3 mg once a week for 12 weeks	Systemic or local treatment - pharmacological agent not specified	15 of 21 patients showed perfect recovery and 4 out of 21 patients showed distinct recovery; The terms “perfect recovery” and “distinct recovery” were not defined by the authors	No side effects were observed
Khan et al.	2014	Open-label	Cutaneous LP	37	3 mg once a week for 6 weeks	Not specified	Improvement in papular eruption, scaly lesions and pigmentation; Complete remission was not observed in any of the enoxaparin-treated patients	8 of 37 patients suffered from local bruises and headache
Iraji et al.	2013	Multicentre randomized clinical study	Disseminated LP	25	5 mg once a week until complete remission or a maximum of 8 weeks	Not specified	8 (32%) patients had complete remission, 10 (40%) patients had partial improvement and 7 patients (28%) had no improvement	No side effects were observed in 24 patients and one patient suffered from a rash of new lesions at the injection site
Lunge et al.	2016	Non-randomized controlled clinical study	Generalized LP	20	5 mg once a week for 16 to 24 weeks	Topical corticosteroid, antihistamines or emollients	13 patients had complete remission of skin lesions. However, 7 patients showed relapse after discontinuation of enoxaparin	No side effects were observed

However, some clinical studies have shown disappointing results. For example, 3 mg subcutaneous enoxaparin was administered once-weekly for a period of 6 weeks to 10 patients with proven LP (with or without oral lesions) (Rai et al., 2002). None of the patients showed clinical remission at the end of the study. Side effects, such as skin necrosis or multiple lesions with increased itching, were observed in 20% of patients not allergic to enoxaparin, heparin, or its derivatives. The observed side effects were severe enough to require discontinuation of enoxaparin. Ferahbas et al. (2003) presented a study in which 7 patients with histologically proven LP were treated with 5 mg of subcutaneous enoxaparin once-weekly for 6 weeks. Mild clinical improvement was observed in only 1 patient and the other 6 patients did not show improvement.

Another study compared the efficacy and safety of methotrexate and enoxaparin in patients with generalized LP, with a 6-month follow up (Lunge et al., 2016). Although 5 mg of subcutaneous enoxaparin administered once weekly for 16–24 weeks produced remission of skin lesions in 13 out of 20 patients, 35% of patients showed relapse after discontinuation of enoxaparin. The authors concluded that methotrexate was not only more effective in terms of causing complete remission of the disease, but also better tolerated and associated with a lower recurrence rate than enoxaparin. An open-labeled, uncontrolled study treated 37 cases of cutaneous LP with 3 mg of enoxaparin once-weekly for 6 weeks. Complete remission was not observed in any of the patients and the reported side effects were local irritation, bruises and headache (Khan et al., 2014). So far, only one randomized clinical trial investigated the efficacy of enoxaparin in patients with disseminated LP (Iraji et al., 2013). In this study, 25 patients were treated with 5 mg of subcutaneous enoxaparin once-weekly for 8 weeks, and 23 patients with 0.5 mg/kg daily oral prednisolone until complete remission was observed or for a maximum of 8 weeks. The reported therapeutic response rate was statistically lower for enoxaparin (32% complete remission) than oral prednisolone (69.6% complete remission). However, the number of patients with reported side effects were non-statistically fewer in the enoxaparin group than the prednisolone group.

## Proposed Reasons for Reported Inconsistent Clinical Outcomes

It is important to note that heparins, including LMWHs such as enoxaparin, have shown inconsistent outcomes when used in other clinical conditions where a non-anticoagulation effect is required. For example, so far 19 clinical studies have investigated the efficacy of heparin or its low molecular weight derivatives in ulcerative colitis (UC), a chronic inflammatory condition (Lean et al., 2015). Some studies reported a significant clinical improvement while others have shown little or no clinical benefit of heparins in this condition. Brazier et al. reported complete remission in 4 of 6 patients with UC when treated with heparin (Brazier et al., 1996). Similarly, 12 out 16 patients with severe refractory UC treated with either intravenous or subcutaneous heparin showed significant clinical improvement (Evans et al., 1997). On the other hand, several randomized studies have reported no clinical benefit of heparin over placebo when used for the management of mild-to-moderate UC (Lean et al., 2015).

One study showed that heparin has an inhibitory effect on methacholine-induced bronchoconstrictive response and, therefore, could potentially be used for the management of asthma (Ceyhan and Celikel, 1995). However, in another study such an effect of heparin was not observed (Pavord et al., 1996).

The possible reasons behind the observed discrepancies in the reported clinical outcomes when enoxaparin was used for the management of LP are briefly discussed below.

### Variable Study Designs

The duration and the dosage regimen of the treatment, and sub-type and severity of the disease could affect the response. For example, clinical studies so far investigating the potential of enoxaparin in LP included the use of: (i) different treatment durations (ranging from 4 to 24 weeks); (ii) different dosage regimens of enoxaparin (e.g., 3 mg once-weekly for 6 weeks or 5 mg once-weekly for 24 weeks, or 30 mg bi-weekly for the first 2 weeks and then 3 mg bi-weekly for the next 4 weeks); (iii) different disease extent and severity (mild, moderate, severe, active or refractory LP); (iv) different forms and types of LP (cutaneous, ulcerative, disseminated, generalized, recalcitrant, localized, hypertrophic, palmoplantar etc.); and (v) inconsistent study end points (remission of disease symptoms, self-reported visual analog scales, histologic improvement etc.). Apart from these explanations, the reported inconsistent clinical outcomes could be due to the (i) use of sub-therapeutic dose of enoxaparin and/or (ii) presence of compositional variation in different batches of enoxaparin.

### Use of Sub-Therapeutic Dose of Enoxaparin

In clinical practice, enoxaparin is normally used for the prevention and treatment of venous thromboembolic disorders, including deep vein thrombosis and pulmonary embolism. The prophylactic dose of enoxaparin in patients with a low-to-moderate risk of venous thromboembolism is 20 mg once daily by subcutaneous injection. For the treatment of venous thromboembolism, the recommended dose of enoxaparin in a 60 kg patient is 90 mg once daily. All the studies, except one, investigating the role of enoxaparin in LP used the dose of either 3 or 5 mg once-weekly (**Table 1**). An earlier report of the successful use of 3 mg subcutaneous enoxaparin in contact dermatitis (Ingber et al., 1994), a T-cell driven inflammatory condition, triggered the use of the sane dose in subsequent studies in patients with LP, also a T-cell mediated autoimmune disease.

Plasma concentrations of enoxaparin after 20 and 40 mg subcutaneous administration are reported to be 1.6 and 3.8 μg/mL, respectively (Sanofi-Aventis Australia Pty Ltd., 2014). Enoxaparin, in various *in vitro*, *in vivo* and *ex vivo* studies, displayed its anti-inflammatory activities at concentrations much higher than its plasma concentrations required to achieve an anticoagulant effect. For example, an *in vivo* study testing the individual non-anticoagulant fragments of enoxaparin for their anti-inflammatory effects used the dose that corresponded to the amount of each fragment present in intact enoxaparin (Lean et al., 2016). The authors reported that the doses for non-anticoagulant fragments were found to be significantly lower than the doses required for their anti-inflammatory effect. In another study, a LMWH was found to be more effective than placebo for treating patients with UC when administrated by extended colon-release tablets (Chande et al., 2010). However, the same benefits were not seen when a LMWH was administered subcutaneously in lower doses (Chande et al., 2010). The immunological activity in LP correlates with the disease severity and the low doses of enoxaparin used in previous studies to treat various forms of LP may not be sufficient to elicit an optimal clinical effect.

### Batch-to-Batch Inconsistency

The reported variable clinical efficacy of enoxaparin when used for the treatment of LP could be because of the batch-to-batch inconsistency in the enoxaparin formulation. Batch-to-batch inconsistency could result in compositional differences between fragments potentially responsible for the anti-inflammatory effects of enoxaparin. The commercially available enoxaparin is standardized only according to the anticoagulant activity; it is not standardized for the non-anticoagulant fragments, which could result in batch-to-batch variations in the presence of these fragments. Patel et al. (2008) developed a capillary electrophoresis method for fingerprinting batches of commercially available enoxaparin. Surprisingly, the method clearly revealed six compositional differences in two different batches of enoxaparin manufactured by the same manufacturer.

The initial study that examined the effects of enoxaparin in LP reported complete regression of the eruption in eight out of the ten patients (Hodak et al., 1998). However, similar outcomes were not observed in some subsequent studies, and it was previously hypothesized that the compositional differences between batches of enoxaparin may be responsible for the inconsistent observations (Ferahbas et al., 2003; Akdeniz et al., 2005).

## Possible Solutions to Avoid Inconsistent Clinical Outcomes

### Determination of Clinical Efficacy of Non-anticoagulant Fragments in LP

We believe that the ideal solution is to obtain non-anticoagulant fragments of enoxaparin, and examine the efficacy of each fragment against LP. There are two principal approaches of obtaining non-anticoagulant fragments of enoxaparin. One approach is through enzymatic or chemical digestion of enoxaparin. Digestion processes, otherwise known as depolymerisation, are often carried out at elevated temperatures. The sulfation pattern of enoxaparin’s fragments is important for the various non-anticoagulant activities (Wang, 2011; Shastri et al., 2015c). At elevated temperatures, some of the fragments undergo desulfation resulting in structural changes and potential loss of their biological activity (Patel et al., 2009c). Enoxaparin undergoes aggregation at low temperatures resulting in the loss of its biological activity (Patel et al., 2009b). Digestion processes also involve freeze-drying and, therefore, the fragments of enoxaparin obtained after digestion may be structurally and therapeutically different from the fragments prior to the digestion process.

Another approach to obtain various fragments of enoxaparin is through chromatographic techniques. Enoxaparin is difficult to separate into its various fragments due to its high polarity, negative charge and structural complexity. However, various chromatographic techniques, such as capillary electrophoresis (Patel et al., 2008), reversed-phase high-performance liquid chromatography (Patel et al., 2009a) and, more recently, ion-exchange chromatography have been developed and validated for the separation of enoxaparin into its fragments (Shastri et al., 2013). The later chromatographic technique successfully separated enoxaparin into more than 20 various fragments without prior chemical or enzymatic digestion of parent enoxaparin. Using the same technique, other studies have identified groups of fragments in enoxaparin with high, low and no anticoagulant activity (Lean et al., 2015, 2016; Shastri et al., 2015c). In addition, Shastri et al. (2013) demonstrated the anti-inflammatory effect of enoxaparin fragments separated through ion-exchange chromatography.

The therapeutic effect of each non-anticoagulant fragment obtained through chromatographic techniques should first be investigated using preclinical animal models of LP. The preclinical testing of non-anticoagulant fragments using an animal model to determine the fragments responsible for the efficacy of enoxaparin in LP is not a prerequisite before evaluating such fragments in humans. However, apart from providing information such as therapeutic efficacy and safety parameters essential to design a clinical study, a preclinical study would provide the dose-response relationship for each non-anticoagulant fragment. It is important to note that all the studies so far looking at the efficacy of enoxaparin in LP have admittedly employed doses that were sub-therapeutic for anticoagulant activity to minimize the risk of bleeding. However, as previously noted, the anti-inflammatory activities of heparin are expressed at higher concentrations, where anticoagulant effects and hence bleeding predominate if anticoagulant fragments are administered. The information on dose-response relationship is crucial as enoxaparin’s fragments have shown dose-dependent anti-inflammatory effects in *ex vivo* and *in vitro* studies (Shastri et al., 2013, 2015b). Enoxaparin is composed of multiple fragments containing the same number of saccharides. For example, enoxaparin contains approximately 6 fragments that are composed of four saccharides (dp4) (Shastri et al., 2013). The relative percentile amount of one of the six dp4 fragments in 500 μg/mL of enoxaparin is approximately 5% (2.5 μg/ml). This fragment, when used at 2.5 μg/mL, was not able to inhibit different types of inflammatory cytokines released from activated human immune cells. However, at 20 μg/mL, the same fragment inhibited the release of IL-4, IL-5, IL-13, and TNF-α by more than 69, 70, 75, and 70%, respectively (Shastri et al., 2015c).

Dose-response activity obtained from an animal model would provide much needed guidance on the likely appropriate dose of active fragments required to elicit therapeutic effects in patients with LP. Therefore, preclinical studies followed by clinical investigations may lead to a new formulation of enoxaparin fragments that exhibit anti-inflammatory properties, without significant anticoagulant activity. The development of such a formulation can therefore serve as a novel therapeutic approach for the treatment of LP. However, development and regulatory approval of such a formulation would require extensive validation and formulation steps that are likely to take a long time to eventuate.

### Monitoring Batch-to-Batch Consistency

Until a novel formulation of non-anticoagulant fragments of enoxaparin is developed and subsequently approved by regulatory agencies and commercially available, studies of different doses of enoxaparin and with large number of patients will provide better insight into the effectiveness of this drug. A key aspect in determining the clinical efficacy of commercially available enoxaparin in LP is the necessity to maintain consistency in the non-anticoagulant fragments between different batches. The best possible way seems to be to obtain the standard chromatographic profiles of non-anticoagulant fragments of a particular batch of enoxaparin with a proven clinical value in LP. Other batches of enoxaparin could then be verified for the batch-to-batch uniformity by comparing the separation patterns of their non-anticoagulant fragments against the standard chromatographic profile.

## Way Forward

### Efficacy of Other LMWHs in LP

Surprisingly, no study so far has investigated the efficacy of unfractionated heparin or LMWHs other than enoxaparin. LMWHs such as dalteparin, tinzaparin, nadroparin, fondaparinax, and reviparin are also approved anticoagulants. Dalteparin and enoxaparin have shown improved clinical outcomes when used for the management of UC (Torkvist et al., 1999; Dotan et al., 2001). On the other hand, no significant beneficial effect was observed with the use of tinzaparin or reviparin in patients with UC (Bloom et al., 2004; de Bievre et al., 2007).

Low-molecular-weight heparins are obtained by enzymatic degradation of heparin or through chemical processes. Currently available LMWHs have different *in vitro* and *ex vivo* non-anticoagulant activities, physico-chemical characteristics and pharmacodynamics. These derivatives also differ in terms of their sulfation pattern, an important parameter for their non-anticoagulant activity, as well as their ability to interact with cellular proteins, endothelial cells and inflammatory cytokines (Prandoni, 2003).

Interestingly, in one study the observed effect of enoxaparin on the release of inflammatory cytokines was opposite to dalteparin (Shastri et al., 2015a,c). Enoxaparin inhibited cytokine release by more than 48%, whereas dalteparin increased their release by more than 25%. Smaller fragments were responsible for the inhibitory effect of enoxaparin and the larger fractions were associated with the stimulatory effect of dalteparin. Therefore, future studies should not only investigate the efficacy of other LMWHs in LP, but also compare the clinical effects of various LMWHs.

### Structural Elucidation of Active Non-anticoagulant Fractions of Enoxaparin

**FIGURE 2:**
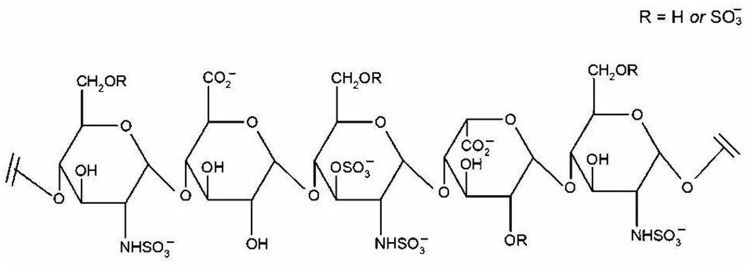
Pentasaccharide sequence of heparin. The binding between this specific sequence and antithrombin III results in the anticoagulant effect of heparin. Reproduced with permission (Patel et al., 2009c).

It is estimated that only 30% of fragments in heparins are composed of the pentasaccharide sequence responsible for their anticoagulant activity. This pentasaccharide sequence selectively binds to anti-thrombin, resulting in enhanced effects of anti-thrombin on factor Xa. The structure of pentasaccharide sequence is shown in **Figure 2**. The fragments of enoxaparin that do not possess anticoagulant activity are reported to be responsible for heparin’s non-anticoagulant activity, including anti-inflammatory effect. Given the potential of enoxaparin in LP, future studies should aim for identification and structural elucidation of therapeutically active non-anticoagulant fragments. After identifying the fragments of enoxaparin and establishing dose-response relationships, their structural elucidation should be carried out using sophisticated techniques such as mass spectrometry and nuclear magnetic resonance. Once the structures of active fragments are established, structure-activity relationships should provide important information on the precise location and sulfation pattern required for the anti-inflammatory activity.

As a new formulation containing identified fragments would have been derived from commercially available enoxaparin, it may face patent and regulatory approval related complications. Elucidation of structure-activity relationships would enable the structural modification of identified fragments without compromising their clinical efficacy in LP. Such an approach would allow the synthesis of new compounds, structurally different from the active non-anticoagulant fragments of enoxaparin, without losing the anti-inflammatory effect. Using a similar approach, an ultra LMWH (known as fondaparinux) was developed and is currently used for the treatment of deep vein thrombosis and acute pulmonary embolism. Fondaparinux is a synthetic pentasaccharide devoid of other non-anticoagulant fragments present in various types of heparins. Therefore, it does not bind to various plasma proteins other than anti-thrombin.

### Mechanism of Action of Active Non-anticoagulant Fractions of Enoxaparin

The mechanisms by which enoxaparin exerts its therapeutic effect in LP is currently unknown. Therefore, future research should investigate the possible mode of action of identified non-anticoagulant fragments of enoxaparin in LP. So far, only one study has investigated the possible mechanisms by which non-anticoagulant fragments of enoxaparin inhibited the release of inflammatory cytokines (Shastri et al., 2015a,c). In this study, the peripheral blood mononuclear cells were collected from asthmatic patients and then the cells were stimulated using lectins, such as phytohaemagglutinin and concanavalin A, in the presence or absence of various non-anticoagulant fragments of enoxaparin. The authors concluded that the potential mechanism by which the tested fragments suppressed the inflammatory response was through directly interacting with cell surface receptors and covering different signaling pathways. However, it remains to be seen if such inhibition is limited to stimulation of cells through plant lectins or is extended to antigen-specific activation of the T cell receptor.

## Conclusion

One of the important reasons for the observed inconsistent clinical outcomes when enoxaparin has been studied for the treatment of LP could be the presence of structurally different non-anticoagulant fragments in different batches of enoxaparin. Based on the available scientific evidence, the authors believe that enoxaparin may play an important role in the management of LP and its potential must be explored using well-designed clinical trials and experimental studies that focus on identifying the anti-inflammatory fragments of enoxaparin and elucidating the mechanism of actions and appropriate therapeutic doses of these non-anticoagulant fragments.

## Author Contributions

RP conceived and designed the review. RP and MS performed the literature search. RP, MS, LM, SZ, and GP analyzed the data and wrote the paper.

## Conflict of Interest Statement

The authors declare that the research was conducted in the absence of any commercial or financial relationships that could be construed as a potential conflict of interest.
